# Preoperative diagnosis, treatment, and outcomes of FEPs of ureters in children: a 13-year retrospective study based on data at a large pediatric medical center

**DOI:** 10.1007/s00345-020-03379-6

**Published:** 2020-08-25

**Authors:** Meng He, Ning Li, Weiping Zhang, Zhentao Ren

**Affiliations:** Department of Urology, Beijing Children’s Hospital, Capital Medical University, National Center for Children’s Health, Nan Li Shi Lu Street No. 56, Beijing, 100045 China

**Keywords:** Feps, Hydronephrosis, Polyps, Pediatric, Ureteral

## Abstract

**Purpose:**

To describe our experience in handling cases of children with fibroepithelial polyps (FEPs) of ureters. We specifically present preoperative diagnosis approaches, provide a clear definition of this entity and its outcomes following treatment.

**Method:**

Clinical data of children with FEPs who were consecutively treated at Beijing Children's Hospital from January 2006 to May 2019 were retrospectively analyzed in this study. The clinical data reviewed included diagnostic, intraoperative, and follow-up data.

**Results:**

Of the 2653 children with surgery for hydronephrosis reviewed, 48 (1.8%) cases of FEPs of the ureters were identified, with a mean age of 109 ± 34.7 months. Among them, males accounted for 95.8%, left side for 81.3%, and proximal ureteral polyps for 97.9%. Notably, 70.8% of patients had only 1 polyp and the median size of the polyps was 2.1 ± 1.8 cm. All patients underwent ultrasound before surgery, which revealed the existence of polyps in 29 (60.4%) children. These polyps were completely resected surgically. The mean follow-up was 82 months (range of 6–153 months) and no cases of recurrences of polyps were seen after surgery during follow-up. The rate of other long-term complications was 9.3%.

**Conclusions:**

In conclusion, FEPs are one of the important causes of hydronephrosis in children. Ultrasound is effective for preoperative diagnosis achieving higher true positive rates than other diagnostic methods. Although the recurrence rate of polyps and symptoms are low after complete resection in children, long-term follow-up is advocated to the adolescence stage to monitor the incidences of urinary tract infections, ureteropelvic junction obstruction and stone formation.

**Electronic supplementary material:**

The online version of this article (10.1007/s00345-020-03379-6) contains supplementary material, which is available to authorized users.

## Introduction

Fibroepithelial polyps (FEPs) of ureters are rare benign tumors in children. They have been linked to the occurrence of hydronephrosis. In a previous study, it was found that 0.5% of children with ureteropelvic junction obstruction developed this condition due to fibroepithelial polyps [1].

Histologically, FEPs are mesodermal tumors characterized by a loose vascular fibrous stroma with an overlying benign transitional epithelium [2]. Symptoms of FEPs include hematuria and flank pain, which are secondary to ureteropelvic junction obstruction [3]. However, in some patients, FEPs are asymptomatic and remain undetected throughout their lives.

In patients with ureteral polyps, it is very important to open the ureter at the correct position during the operation, rather than blindly performing completely dismembered UPJ before exposure of polyp base. Therefore, if FEPs can be diagnosed preoperatively, it will be instructive for surgery. In a traditional view, it is very difficult to diagnose FEPs preoperatively. As a large children's medical center in northern China, a considerable number of children with FEPs have been treated in our center over the past decade. We observed that the preoperative diagnosis rate of this condition is relatively very high. In this study, we describe our experience in handling children with FEPs of the ureters. We specifically shed light on preoperative diagnosis approaches, provide a clear definition of this entity and its prognosis following treatment.

## Materials and methods

Clinical data of children with FEPs who were consecutively treated in Beijing Children's Hospital from January 2006 to May 2019 was retrospectively reviewed in this study. The clinical data analyzed included: diagnostic approaches, intraoperative, and follow-up data. To perform intravenous urography (IVU), abdominal plain films were performed at 10, 20, and 40 min following contrast injection. For all ultrasound studies, the children were fasted for 8 h, and then drank adequate amounts of water (about 500–1000 ml) for 30 min to cause diuresis and dilate the renal pelvis to enhance detection of obstruction and visualization of fibroepithelial polyps [4]. The diagnosis of FEPs was based on intraoperative direct vision findings and histology. Surgery was performed by pediatric urologists with more than 5 years of clinical experience. All patients were followed up for more than 6 months after the operation. During the follow-up, IVU and ultrasound were performed.

## Results

In a period of 13 years, 2653 children received surgery for hydronephrosis in our center. Polyps of the ureter were identified as the cause of hydronephrosis in 48 (1.8%) patients. The mean age of the children was 109 ± 34.7 months. Among them, males accounted for 95.8%, left side for 81.3%, and proximal ureteral polyps for 97.9%. Notably, 70.8% of patients had only 1 polyp and the median size of the polyps was 2.1 ± 1.8 cm. Most of the children with hydronephrosis caused by ureteral polyps presented with flank pain and/or hematuria. One patient presented with a fever urinary tract infection and four patients were asymptomatic. The rest of the patients’ characteristics are shown in Table 1.

**Table 1:** Patients characteristics

Age (months)	109 ± 34.7
*Gender*	
Male	46 (95.8%)
Female	2 (4.1%)
*Side*	
Left	39 (81.3%)
Right	6 (12.5%)
Bilateral	3 (6.2%)
*Location*	
Proximal ureter	47 (97.9%)
Middle ureter	1 (2.1%)
Distal ureter	0
Size(cm)	2.1 ± 1.8
*No. polyps*	
1	34 (70.8%)
2	6 (12.5%)
3	1 (2.1%)
≥ 4	7 (14.6%)
*Symptoms*	
Flank pain	39 (81.3%)
Hematuria	9 (18.8%)
Urinary tract infection	1 (2.1%)
No symptoms	4 (8.3%)
Hydronephrosis	48 (100%)

Values are presented as mean ± standard deviation

**Fig. 1:**
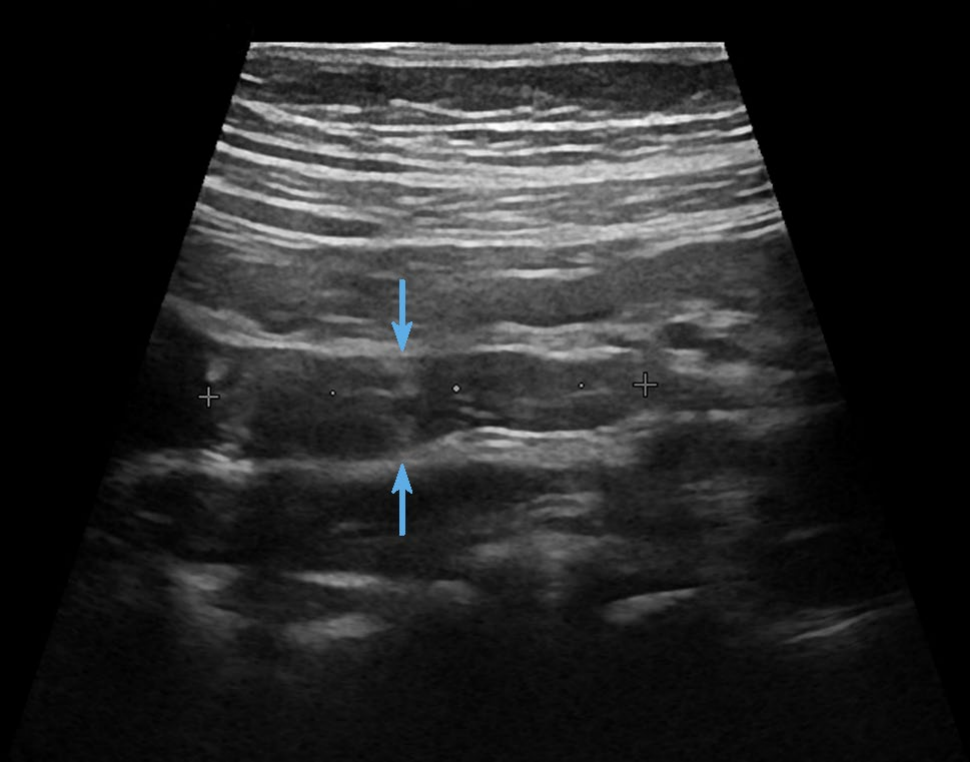
11-Year-old boy with intermittent abdominal pain, longitudinal sonogram reveals a mildly echogenic mass at the proximal ureter (arrows)

**Fig. 2:**
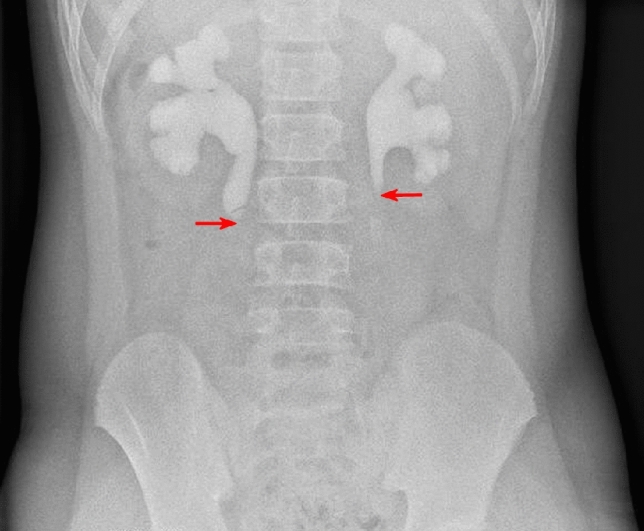
IVP of a 11-year-old boy with bilateral ureteral polyps showing filling defects (red arrows) in bilateral proximal ureter

**Fig. 3:**
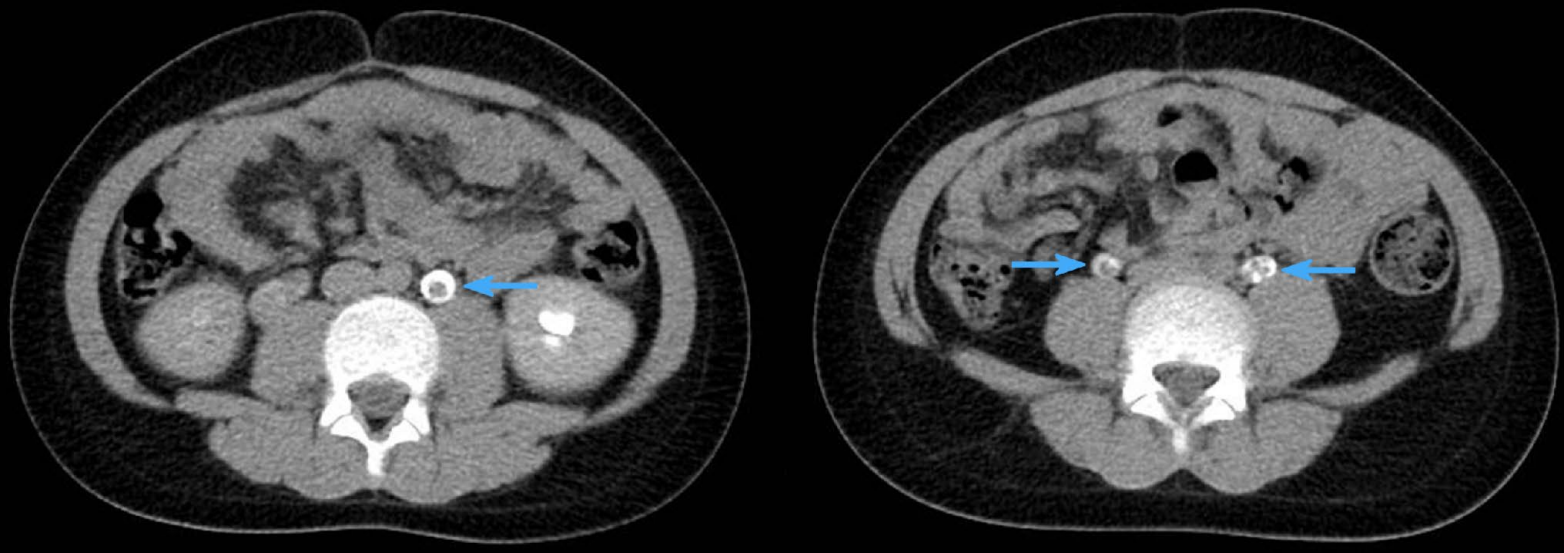
Delayed contrast enhanced CT scan of a 10-year-old boy with bilateral ureteral polyps showing filling defects (blue arrows) in bilateral proximal ureter

All 48 patients underwent ultrasound before surgery and this identified polyps in 29 (60.4%) of the children (Fig. 1). Next, IVU was performed in 41 (85.4%) children and the positive rate of preoperative FEPs diagnosis was 34.1% (Fig. 2). For 6 children who received an abdominal CT scan, the positive rate was 50% (Fig. 3). For details see attached Tables 2.

.

**Table 2:** Preoperative diagnosis with image

Image	Total	Positive	Negative
Ultrasound	48 (100%)	29 (60.4%)	19 (39.6%)
Intravenous urography (IVU)	41 (85.4%)	14 (34.1%)	27 (65.9%)
Computed tomography (CT)	6 (12.5%)	3 (50%)	3 (50%)

Open dismembered pyeloplasty was performed in 26 (54.1%) children, and other patients underwent laparoscopic dismembered pyeloplasty (35.4%), laparoscopic ureteroureterostomy (2.1%) and open ureteroureterostomy (8.3%). The full treatment data is shown in Table 3.

**Table 3:** Treatment and outcome

*Treatment*	
Laparoscopic ureteroureterostomy	17 (35.4%)
Open dismembered pyeloplasty	1 (2.1%)
Laparoscopic dismembered pyeloplasty	26 (54.2%)
Open ureteroureterostomy	4 (8.3%)
*Outcome*	
Recurrence of ureteral polyp	0 (0%)
Urinary tract infection (UTI)	1 (2.3%)
Urinary calculi	2 (4.7%)
Ureteropelvic junction obstruction (UPJO)	1 (2.3%)

The mean follow-up duration was 82 months (range 6–153). Forty-three patients were followed up by telephone, and 25 patients visited our hospital for an ultrasound examination. The rate of complications was 9.3%, and no recurrence of ureteral polyps was found in all patients. A 12-year-old patient had poor renal function before an operation, so the time of nephrostomy was prolonged after surgery (three months in total). It is hoped that this measure can make his renal function recover as much as possible. After the removal of a nephrostomy tube, the child developed abdominal pain and fever. Routine urine test and blood test showed that it is caused by urinary tract infection. After regular anti-inflammatory treatment, the symptoms were relieved. During the telephone follow-up, this patient reported the disappearance of symptoms, and no recurrence of polyp was found based on the ultrasound performed 6 months after surgery. Another male patient, who was operated at the age of 9, had hematuria at the age of 16, which was considered a proximal ureteral stone. He had obstruction of the ureteropelvic junction which was resolved by a repeat pyeloplasty. Another patient had a stone in the renal pelvis 15 months after the operation, without symptoms such as hematuria and abdominal pain. The patient was advised to exercise and drink enough water frequently. Upon reexamination, no stone was found. In another patient, a similar renal pelvis stone was found 4 years after the operation. After extracorporeal shock wave lithotripsy in another hospital, the stone had disappeared. The outcome data of all patients are shown in Table 3.

## Discussion

Ureteral tumors are very rare in children. In a previous study, it was found that 0.5% of children with ureteropelvic junction obstruction developed this condition due to fibroepithelial polyps [1]. However, they are the most common benign mesodermal tumors of the urinary tract [5]. In the current study, the incidence of FEPs of the ureters in children with surgery for hydronephrosis was 1.8%. In another study, the incidence was 4.5%, which is higher than that of this study. But it should be noted that in their study, 60% of patients had multiple polyps or filiform, and 40% had single or bilobed types, which is inconsistent with our conclusion. This may be explained by the fact that only 15 cases of fibroepithelial polyps of the ureter were included in their study [6].

The pathogenesis of FEPs of the ureters is still unknown. Adult urologists believe that factors such as obstruction, trauma, irritation, infection, exogenous carcinogens, hormone imbalance, and allergy may be possible pathogenic causes [7]. However, given the differences in epidemiological characteristics between adults and children, we believe that the pathogenesis of FEPs in childhood may differ from that in adults. Ludwig et al. reviewed 68 studies on adult FEPs in 2015. They found that, of the 131 patients with available data, 71 were female (55.9%) and polyps were evenly distributed among the left and right ureter [7]. In the current study, males accounted for 95.9%, left-side prevalence for 81.6%, and proximal ureteral polyps for 98.0%. Li et al. obtained similar conclusions and found that there is a predilection for males (92.0%) and left side ureter (67.0%) [8]. On the other hand, the pathogenesis of ureteropelvic junction obstruction (UPJO) in children is still unclear. Moreover, there exist conflicting reports on the etiopathogenesis of UPJO, with several studies showing decreased ICC and increased collagen in the narrow segment [9]. To our knowledge, no specific cause of ureteral polyps has been established, and this requires further exploration.

Previous studies show that the preoperative diagnosis of ureteral fibroepithelial polyps is relatively difficult due to the symptomatic and radiographic similarity of this condition to intrinsic UPJ obstruction. In three more-recent case series, the incidence of filling defects varied between 0.0% and 27.0% for IVP and 21.0% for magnetic resonance urography [8]. In our center, the preoperative diagnosis of hydronephrosis was mainly based on ultrasound and IVP and CT. MRI and intraoperative retrograde pyelogram are not routinely performed for children. Notably, our preoperative diagnosis rate of FEPs is higher than that reported in the literature, with the positive rate of ultrasound reaching 61.2%. Duplex Doppler sonography revealed masses of mildly echogenic and non-shadowing with well-defined margins outlined by a urine-distended pelvis and blood flow. In addition, the renal pelvis appeared dilated while the ureter appeared nondilated [4]. We believe that the false-negative rate is due to limited experience, and an experienced pediatric ultrasound doctor can easily diagnose more than half of ureteral polyps. Therefore, we do not recommend preoperative CT or MRI examination for children suspected of hydronephrosis caused by FEPs, but propose ultrasound for preoperative evaluation.

Some incidences of ureteral concomitant malignancy occur in adults. Concomitant transitional cell carcinoma was reported in 1/134 adult with ureteral polyps. However, malignant tumors are even rarer in children. In the course of this case review, only one case was pathologically diagnosed as urothelial papilloma. Although the possibility of a malignant tumor is low, we still recommend open surgery or laparoscopic surgery for polypectomy in children, instead of ureteroscopy. This is because the majority of FEPs in children are located in the proximal ureter (97.9%), which is easier to remove by surgery. Furthermore, given the age of patients, ureteroscopy is limited by the difficulty of reaching the proximal ureter. Recurrence of polyps is also likely causing ureteral stenosis. Once major concern for surgeons is the occurrence of stricture after resection due to the long ureteral defect which increases the tension of the anastomosis. We recommend that, if FEPs are diagnosed preoperatively, during the operation, the peristalsis of the proximal ureter should be observed first, and the ureter should be opened longitudinally at the position suspected to be the polyp base, instead of complete dismember UPJ before the basal part of polyp is exposed. Dai et al. stated that if the ureter is opened longitudinally, and the distance from the pedicles to the UPJ is shorter than 2 cm, pyeloplasty might be feasible [3]. In this study, tension-free anastomosis was achieved in all patients, including those with a wide polyp base.

The prognosis of FEPs seems to be good. In most patients, no abdominal pain, hematuria, and other symptoms were recorded after surgery, and no recurrence of the ureteral polyp was found after complete resection. Of the 4 children with complications in this study, three developed complications more than one year after surgery. We, therefore, believe long-term follow-up is crucial to assess the occurrence of urinary tract infections, ureteropelvic junction obstruction, and stone formation.

## Conclusion

In conclusion, FEPs are important causes of hydronephrosis in children. Ultrasound is effective for preoperative diagnosis achieving higher true positive rates than other diagnostic methods. Although the recurrence rate of polyps and symptoms are low after complete resection in children, long-term follow-up is advocated to the adolescence stage to monitor the incidences of urinary tract infections, ureteropelvic junction obstruction, and stone formation.

## Electronic supplementary material

Below is the link to the electronic supplementary material.

Supplementary file1 (PDF 106 kb)
