# Supplementary material for: Preoperative diagnosis, treatment, and outcomes of FEPs of ureters in children: a 13-year retrospective study based on data at a large pediatric medical center
Source: World J Urol. 2020 Aug 25;39(6):2239–43. doi: 10.1007/s00345-020-03379-6 (PMC8217005; doi:10.1007/s00345-020-03379-6)
Supplement: Supplementary file 1 — Supplementary file1 (PDF 106 kb) [file 345_2020_3379_MOESM1_ESM.pdf]

Preoperative Diagnosis, Treatment and Outcomes of FEPs of Ureters in Children: A 13-Year Retrospective Study Based on Data at a Large Pediatric Medical Center

Introduction

Fibroepithelial polyps (FEPs) of ureters are rare benign tumors in children. They have been linked to the occurrence of hydronephrosis. In a previous study, it was found that 0.5% of children with ureteropelvic junction obstruction developed this condition due to fibroepithelial polyps[1].

Histologically, FEPs are mesodermal tumors characterized by a loose vascular fibrous stroma with an overlying benign transitional epithelium[2]. Symptoms of FEPs include hematuria and flank pain, which are secondary to ureteropelvic junction obstruction[3]. However, in some patients, FEPs are asymptomatic and remain undetected throughout their lives.

In patients with ureteral polyps, it is very important to open the ureter at the correct position during the operation, rather than blindly performing completely dismembered UPJ before exposure of polyp base. Therefore, if FEPs can be diagnosed preoperatively, it will be instructive for surgery. In traditional view, it is very difficult to diagnose FEPs preoperatively. As a large children's medical center in northern China, a considerable number of children with FEPs have been treated in our center over the past decade. We observed that the preoperative diagnosis rate of this condition is relatively very high. In this study, we describe our experience in handling children with FEPs of the ureters. We specifically shed light on preoperative diagnosis approaches, provide a clear definition of this entity and its prognosis following treatment.

Materials and methods

Clinical data of children with FEPs who were consecutively treated in Beijing Children's Hospital from January 2006 to May 2019 was retrospectively reviewed in this study. The clinical data analyzed included: diagnostic approaches, intraoperative and follow-up data. To perform intravenous urography (IVU), abdominal plain films were performed at 10, 20, and 40 min following contrast injection. For all ultrasound studies, the children were fasted for 8 h, and then drank adequate amounts of water (about 500-1,000 ml) for 30 min to cause diuresis and dilate the renal pelvis to enhance detection of obstruction and visualization of fibroepithelial polyps[4]. The diagnosis of FEPs was based on intraoperative direct vision findings and histology. Surgery was performed by pediatric urologists with more than 5 years of clinical experience. All patients were followed up for more than 6 months after

operation. During the follow-up, IVU and ultrasound were performed.

Results

In a period of 13 years, 2653 children received surgery for hydronephrosis in our center. Polyps of the ureter were identified as the cause of hydronephrosis in 48 (1.8%) patients. The mean age of the children was 109±34.7 months. Among them, male accounted for 95.8%, left side for 81.3%, and proximal ureteral polyps for 97.9%. Notably, 70.8% of patients had only 1 polyp and the median size of the polyps was 2.1±1.8 cm. Most of the children with hydronephrosis caused by ureteral polyps presented with flank pain and/or hematuria. One patient presented with fever urinary tract infection and four patients were asymptomatic. The rest of the patients' characteristics are shown in Table 1.

All 48 patients underwent ultrasound before surgery, and this identified polyps in 29 (60.4%) of the children (Fig 1). Next, IVU was performed in 41 (85.4%) children and the positive rate of preoperative FEPs diagnosis was 34.1% (Fig 2). For 6 children who received abdominal CT scan, the positive rate was 50% (Fig 3). For details see attached Tables 2.

Open dismembered pyeloplasty was performed in 26 (54.1%) children, and other patients underwent laparoscopic dismembered pyeloplasty (35.4%), laparoscopic ureteroureterostomy (2.1%) and open ureteroureterostomy (8.3%). The full treatment data is shown in Table 3.

Mean follow-up duration was 82 months (range 6 to 153). Forty-three patients were followed up by telephone, and 25 patients visited our hospital for ultrasound examination. The rate of complications was 9.3%, and no recurrence of ureteral polyps was found in all patients. A 12-year-old patient had poor renal function before operation, so the time of nephrostomy was prolonged after surgery (three months in total). It is hoped that this measure can make his renal function recover as much as possible. After the removal of nephrostomy tube, the child developed abdominal pain and fever. Routine urine test and blood test showed that it is caused by urinary tract infection. After regular anti-inflammatory treatment, the symptoms were relieved. During the telephone follow-up, this patient reported disappearance of symptoms, and no recurrence of polyp was found based on the ultrasound performed 6 months after surgery. Another male patient, who was operated at the age of 9, had hematuria at the age of 16, which was considered a proximal ureteral stone. He had obstruction of the ureteropelvic junction which was resolved by a repeat pyeloplasty. Another patient had a stone in the renal pelvis 15 months after operation, without symptoms such as hematuria and abdominal pain. The patient was

advised to exercise and drink enough water frequently. Upon reexamination, no stone was found. In another patient, a similar renal pelvis stone was found 4 years after operation. After extracorporeal shock wave lithotripsy in another hospital, the stone had disappeared. The outcome data of all patients is shown in Table 3.

Discussion

Ureteral tumors are very rare in children. In a previous study, it was found that 0.5% of children with ureteropelvic junction obstruction developed this condition due to fibroepithelial polyps[1]. However, they are the most common benign mesodermal tumors of the urinary tract[5]. In the current study, the incidence of FEPs of the ureters in children with surgery for hydronephrosis was 1.8%. In another study, the incidence was 4.5%, which is higher than that of this study. But it should be noted that in their study, 60% of patients had multiple polyps or filiform, and 40% had single or bilobed types, which is inconsistent with our conclusion. This may be explained by the fact that only 15 cases of fibroepithelial polyps of the ureter were included in their study[6].

The pathogenesis of FEPs of the ureters is still unknown. Adult urologists believe that factors such as obstruction, trauma, irritation, infection, exogenous carcinogens, hormone imbalance and allergy may be possible pathogenic causes[7]. However, given the differences in epidemiological characteristics between adults and children, we believe that the pathogenesis of FEPs in childhood may differ from that in adults. Ludwig et al. reviewed 68 studies on adult FEPs in 2015. They found that, of the 131 patients with available data, 71 were female (55.9%) and polyps were evenly distributed among the left and right ureter[7]. In the current study, male accounted for 95.9%, left side prevalence for 81.6%, and proximal ureteral polyps for 98.0%. Li et al. obtained similar conclusions and found that there is a predilection for males (92.0%) and left side ureter (67.0%)[8]. On the other hand, the pathogenesis of ureteropelvic junction obstruction (UPJO) in children is still unclear. Moreover, there exists conflicting reports on the etiopathogenesis of UPJO, with several studies showing decreased ICC and increased collagen in the narrow segment[9]. To our knowledge, no specific cause of ureteral polyps has been established, and this requires further exploration.

Previous studies show that the preoperative diagnosis of ureteral fibroepithelial polyps is relatively difficult due to the symptomatic and radiographic similarity of this condition to intrinsic UPJ obstruction. In three more-recent case series, the incidence of filling defects varied between 0.0% and

27.0% for IVP and 21.0% for magnetic resonance urography[8]. In our center, preoperative diagnosis of hydronephrosis was mainly based on ultrasound and IVP and CT. MRI and intraoperative retrograde pyelogram are not routinely performed for children. Notably, our preoperative diagnosis rate of FEPs is higher than that reported in the literature, with the positive rate of ultrasound reaching 61.2%. Duplex Doppler sonography revealed masses of mildly echogenic and non-shadowing with well-defined margins outlined by a urine-distended pelvis and blood flow. In addition, the renal pelvis appeared dilated while the ureter appeared nondilated[4]. We believe that the false negative rate is due to limited experience, and an experienced pediatric ultrasound doctor can easily diagnose more than half of ureteral polyps. Therefore, we do not recommend preoperative CT or MRI examination for children suspected of hydronephrosis caused by FEPs, but propose ultrasound for preoperative evaluation.

Some incidences of ureteral concomitant malignancy occur in adults. Concomitant transitional cell carcinoma was reported in 1/134 adult with ureteral polyps. However, malignant tumors are even rarer in children. In the course of this case review, only one case was pathologically diagnosed as urothelial papilloma. Although the possibility of malignant tumor is low, we still recommend open surgery or laparoscopic surgery for polypectomy in children, instead of ureteroscopy. This is because the majority of FEPs in children are located in the proximal ureter (97.9%), which is easier to remove by surgery. Furthermore, given the age of patients, ureteroscopy is limited by the difficulty of reaching the proximal ureter. Recurrence of polyps is also likely causing ureteral stenosis. Once major concern for surgeons is the occurrence of stricture after resection due to the long ureteral defect which increases tension of the anastomosis. We recommend that, if FEPs are diagnosed preoperatively, during the operation, the peristalsis of the proximal ureter should be observed first, and the ureter should be opened longitudinally at the position suspected to be the polyp base, instead of complete dismember UPJ before the basal part of polyp is exposed. Dai et al. stated that if the ureter is opened longitudinally, and the distance from the pedicles to the UPJ is shorter than 2 cm, pyeloplasty might be feasible[3]. In this study, tension-free anastomosis was achieved in all patients, including those with a wide polyp base.

The prognosis of FEPs seems to be good. In most patients, no abdominal pain, hematuria and other symptoms were recorded after surgery, and no recurrence of ureteral polyp was found after complete resection. Of the 4 children with complications in this study, three developed complications more than one year after surgery. We therefore believe long-term follow-up is crucial to assess the occurrence of

urinary tract infections, ureteropelvic junction obstruction and stone formation.

Conclusion

In conclusion, FEPs is an important causes of hydronephrosis in children. Ultrasound is effective for preoperative diagnosis achieving higher true positive rates than other diagnostic methods. Although the recurrence rate of polyps and symptoms are low after complete resection in children, long-term follow-up is advocated to adolescence stage to monitor the incidences of urinary tract infections, ureteropelvic junction obstruction and stone formation.

Conflicts of Interest:

None of the contributing authors have any conflict of interest, including specific financial interests or relationships and affiliations relevant to the subject matter or materials discussed in the manuscript.

Authors' Contribution

Meng He (first author): Protocol development, Data analysis, Manuscript writing

Ning Li (Co-corresponding Author): Protocol development, Manuscript editing

Weiping Zhang (Co-corresponding Author): Protocol development, Manuscript editing

Zhentao Ren: Data collection

References:

1. Kara C, Resorlu B, Oguz U, Unsal A. Incidentally detected ureteral fibroepithelial polyps in children: is endoscopic treatment of them really necessary? INT UROL NEPHROL. 2010;42(1):1-5. 'doi:'10.1007/s11255-009-9567-4.

2. Adey GS, Vargas SO, Retik AB, Borer JG, Mandell J, Hendren WH et al. Fibroepithelial polyps causing ureteropelvic junction obstruction in children. J Urol. 2003;169(5):1834-6. 'doi:'10.1097/01.ju.0000061966.21966.94.

3. Dai LN, Chen CD, Lin XK, Wang YB, Xia LG, Liu P et al. Retroperitoneal laparoscopy management for ureteral fibroepithelial polyps causing hydronephrosis in children: a report of five cases. J PEDIATR UROL. 2015;11(5):251-7. 'doi:'10.1016/j.jpurol.2015.02.019.

4. Wang XM, Jia LQ, Wang Y, Wang N. Utilizing ultrasonography in the diagnosis of pediatric

fibroepithelial polyps causing ureteropelvic junction obstruction. PEDIATR RADIOL. 2012;42(9):1107-11. 'doi:'10.1007/s00247-012-2404-4.

5. Williams TR, Wagner BJ, Corse WR, Vestevich JC. Fibroepithelial polyps of the urinary tract. ABDOM IMAGING. 2002;27(2):217-21. 'doi:'10.1007/s00261-001-0066-z.

6. Babu R, Vittalraj P, Sundaram S, Shalini S. Pathological changes in ureterovesical and ureteropelvic junction obstruction explained by fetal ureter histology. J PEDIATR UROL. 2019;15(3):240-1. 'doi:'10.1016/j.jpurol.2019.02.001.

7. Niu ZB, Yang Y, Hou Y, Chen H, Wang CL. Ureteral polyps: an etiological factor of hydronephrosis in children that should not be ignored. PEDIATR SURG INT. 2007;23(4):323-6. 'doi:'10.1007/s00383-007-1884-z.

8. Ludwig DJ, Buddingh KT, Kums JJ, Kropman RF, Roshani H, Hirdes WH. Treatment and outcome of fibroepithelial ureteral polyps: A systematic literature review. Can Urol Assoc J. 2015;9(9-10):E631-7. 'doi:'10.5489/cuaj.2878.

9. Li R, Lightfoot M, Alsyouf M, Nicolay L, Baldwin DD, Chamberlin DA. Diagnosis and management of ureteral fibroepithelial polyps in children: a new treatment algorithm. J PEDIATR UROL. 2015;11(1):21-2. 'doi:'10.1016/j.jpurol.2014.08.004.
